# Associations between exposure to heavy metal and sarcopenia prevalence: a cross-sectional study using NHANES data

**DOI:** 10.3389/fpubh.2025.1588041

**Published:** 2025-07-04

**Authors:** Yingying Zhang, Qianbing Li, Xiangfei Wang

**Affiliations:** School of Journalism and Communication, Wuhan Sports University, Wuhan, China

**Keywords:** sarcopenia, heavy metal exposure, NHANES, machine learning, SHAP

## Abstract

**Background:**

Sarcopenia is a condition that adversely affects individuals’ quality of life and physical health. Exposure to heavy metals poses a significant risk to human health; however, the impact of heavy metal exposure on sarcopenia remains unclear. Therefore, this study expects to construct a risk prediction machine model of heavy metal exposure on sarcopenia and to interpret and analyze it.

**Methods:**

Model construction was based on data from the NHANES database, covering the years 2011 to 2018. The predictor variables included BA, CD, CO, CS, MN, MO, PB, SB, SN, TL, and W. Additionally, demographic characteristics and health factors were included in the study as confounders. After identifying the core variables, optimal machine learning models were constructed, and SHAP analyses were performed.

**Results:**

We found that the LGBM model exhibited the best predictive performance. SHAP analysis revealed that TL, SN, and CS negatively influenced the prediction of sarcopenia, while CD positively contributed to it. Additionally, le8 BMI was the covariate that had the most significant positive impact on the prediction of sarcopenia in our model.

**Conclusion:**

For the first time, we have developed a machine learning (ML) model to predict sarcopenia based on indicators of heavy metal exposure. This model has accurately identified a series of key factors that are strongly associated with sarcopenia induced by heavy metal exposure. We can now identify individuals at an early stage who are suffering from sarcopenia due to heavy metal exposure, thereby reducing the physical and economic burden on public health.

## 1 Introduction

Sarcopenia is a concept introduced in 1988, which refers to the presence of low muscle mass with low muscle function. In the context of the increasingly severe aging population, sarcopenia, an age-related geriatric syndrome, is progressively emerging as a condition that adversely affects the life, health, and quality of life of older adults. Some studies indicate that the prevalence of sarcopenia ranges from 8 to 36% in individuals under 60 years of age and from 10 to 27% in those over 60 years of age (1). While sarcopenia itself poses risks of mobility problems, falls and even bone fractures (2), some researchers in recent years have also found a strong link with liver disease (3), cardiovascular disease (4) and even cancer (5). This indicates that, in addition to the health risks directly associated with sarcopenia, there is a substantial likelihood that it can contribute to an increased risk of other diseases, as well as a combination of conditions that may exacerbate overall health threats. Furthermore, sarcopenia can lead to heightened healthcare costs, with patients incurring significantly higher expenses for hospitalization, in-home care, and other related services (6). Therefore, in light of these numerous risks, the European Working Group on Sarcopenia in Older People 2 (EWGSOP2) urges professionals to take proactive measures and commit to the early detection and treatment of sarcopenia. This approach aims to mitigate the financial burden and physical harm associated with the advanced stages of the condition (7).

In addition to primary sarcopenia, which is associated with aging, secondary sarcopenia, resulting from other diseases, has also emerged as a significant risk factor (8). More specifically, sarcopenia has been identified as resulting from a complex interplay of interdependent pathophysiological mechanisms, including aging, physical inactivity, neuromuscular injury, postprandial anabolic resistance, insulin resistance, lipotoxicity, endocrine factors, oxidative stress, mitochondrial dysfunction, and inflammation (9). It is common for patients with neurological critical illnesses to experience reduced muscle mass, decreased strength, and neurological impairment. Both muscle atrophy and muscle weakness can lead to a decline in physical function (10). It has been found that oxidative stress, resulting from an imbalance between the production of reactive oxygen species and antioxidant defenses, contributes to the development of sarcopenia (11). There is a significant relationship between inflammation and oxidative stress, where inflammation can result from oxidative stress-induced redox imbalance and the sustained upregulation of pro-inflammatory mediators (12). The rapid loss of muscle mass and strength is primarily attributed to excessive protein catabolism (13). Inflammatory cytokines activate numerous molecular pathways involved in skeletal muscle atrophy during inflammation, resulting in an imbalance between protein synthesis and catabolism, which subsequently affects sarcopenia (14). Additionally, the effects of environmental pollution on health are closely linked to age. Age is a significant factor influencing serum chemical levels, and the accumulation of chemicals in human serum tends to increase with advancing age (15). Of particular concern is the risk of heavy metal exposure. Such exposure can jeopardize biological functions and growth, potentially leading to the development of various serious diseases when metals accumulate in one or more organs (16). Due to the significant negative effects of heavy metal exposure on human health, there has been an increase in contemporary studies aimed at identifying the specific detrimental impacts of heavy metals on the human body, as well as potential countermeasures. Existing research indicates that health issues associated with heavy metal exposure may include cardiovascular disease, neurological damage, kidney impairment, and an elevated risk of cancer and diabetes. The prevailing theory regarding the mechanism of heavy metal toxicity in humans, resulting from excessive intake, suggests that it involves the production of reactive oxygen species, which lead to oxidative damage and various health-related adverse effects (17). Mercury, lead, chromium, cadmium, and arsenic are the most common heavy metals that cause toxicity in humans. They lead to the production of reactive oxygen species (ROS), weaken antioxidant defenses, inactivate enzymes, and contribute to oxidative stress (18). Among the primary effects of mercury-induced toxicity are the inhibition of antioxidant defenses, alterations in the oxidant-antioxidant balance, and an increase in reactive oxygen species (ROS). These effects are associated with methylmercury, a neurotoxic compound that leads to lipid peroxidation, mitochondrial damage, microtubule disruption, and the accumulation of neurotoxic molecules (19). Manganese accumulation primarily occurs in the basal ganglia and can lead to manganese toxicity syndrome, which presents symptoms of cognitive dysfunction and motor impairment similar to those seen in Parkinson’s disease (PD) (20).

From this, we identified a complex relationship between the numerous negative effects of heavy metal exposure and the mechanisms underlying sarcopenia. These mechanisms are associated with protein synthesis, mitochondrial damage, oxidative stress, biometabolism, neuronal damage, and various chronic diseases. However, despite the numerous potential molecular and pathological connections between heavy metal exposure and sarcopenia, there remains a significant gap in direct research examining the relationship between these two factors. While the existing literature primarily addresses the independent toxic effects of heavy metals or focuses on a singular cause of sarcopenia, there is a notable scarcity of systematic studies investigating the interactions between them. This is particularly true regarding large epidemiological samples and comprehensive molecular mechanistic analyses. This gap restricts our understanding of the specific role that heavy metal exposure plays in the development of sarcopenia and impedes the formulation of targeted prevention or intervention strategies.

Currently, studies of relatively complex linear or non-linear relationships have shown that multiple variables play a role, making it difficult for traditional statistical methods to capture the correlations and clarify the relationships. In this regard, we have found that machine learning is able to capture such complex correlation patterns and identify potential relationships between different elements through its powerful feature selection and non-linear modeling capabilities. The process of machine learning (ML) involves learning from data and typically employs concepts from optimization theory and numerical analysis to tackle specific problems. By focusing on the mathematical structures involved, we can achieve varying levels of adaptability for *post hoc* visualization and interpretation, while also balancing trade-offs between computational complexity, data volume, and performance (21). The use of ML is more mature in medical applications, including drug discovery and development (22), structural health monitoring (23), and medical imaging (24). In terms of disease prediction, one-class logistic regression (OCLR) models have been used to find correlations between tumor stem cells and immune checkpoint expression and infiltrating immune cells (25). SHapley Additive exPlanations (SHAP) is a widely used method for interpreting machine learning models, providing detailed insights into model predictions by illustrating the contribution of each feature to the overall output. In this study, SHAP effectively identifies heavy metal-related features that are significantly associated with the risk of sarcopenia, while eliminating variables that contribute weakly or interfere with the model. This approach results in a streamlined and efficient model, enhancing the accuracy and speed of subsequent predictions, mitigating issues such as overfitting, and improving the model’s generalization capabilities.

Therefore, we sought to explore the relationship between heavy metal exposure and sarcopenia by examining various factors associated with heavy metal exposure. Utilizing data from the NHANES database, we constructed an accurate prediction model for sarcopenia by incorporating information related to heavy metal exposure, demographic characteristics, and health factors. This model employed multiple ML algorithms and SHAP interpretable machine learning techniques to identify which heavy metal exposures significantly contribute to the risk of sarcopenia. By highlighting the detrimental effects of heavy metal exposure, we present new opportunities for early screening and risk prediction of sarcopenia. This proactive approach aims to mitigate the potential health risks associated with the interplay between heavy metal exposure and sarcopenia through timely disease onset screening.

## 2 Methodology

### 2.1 Study population

**Figure 1:**
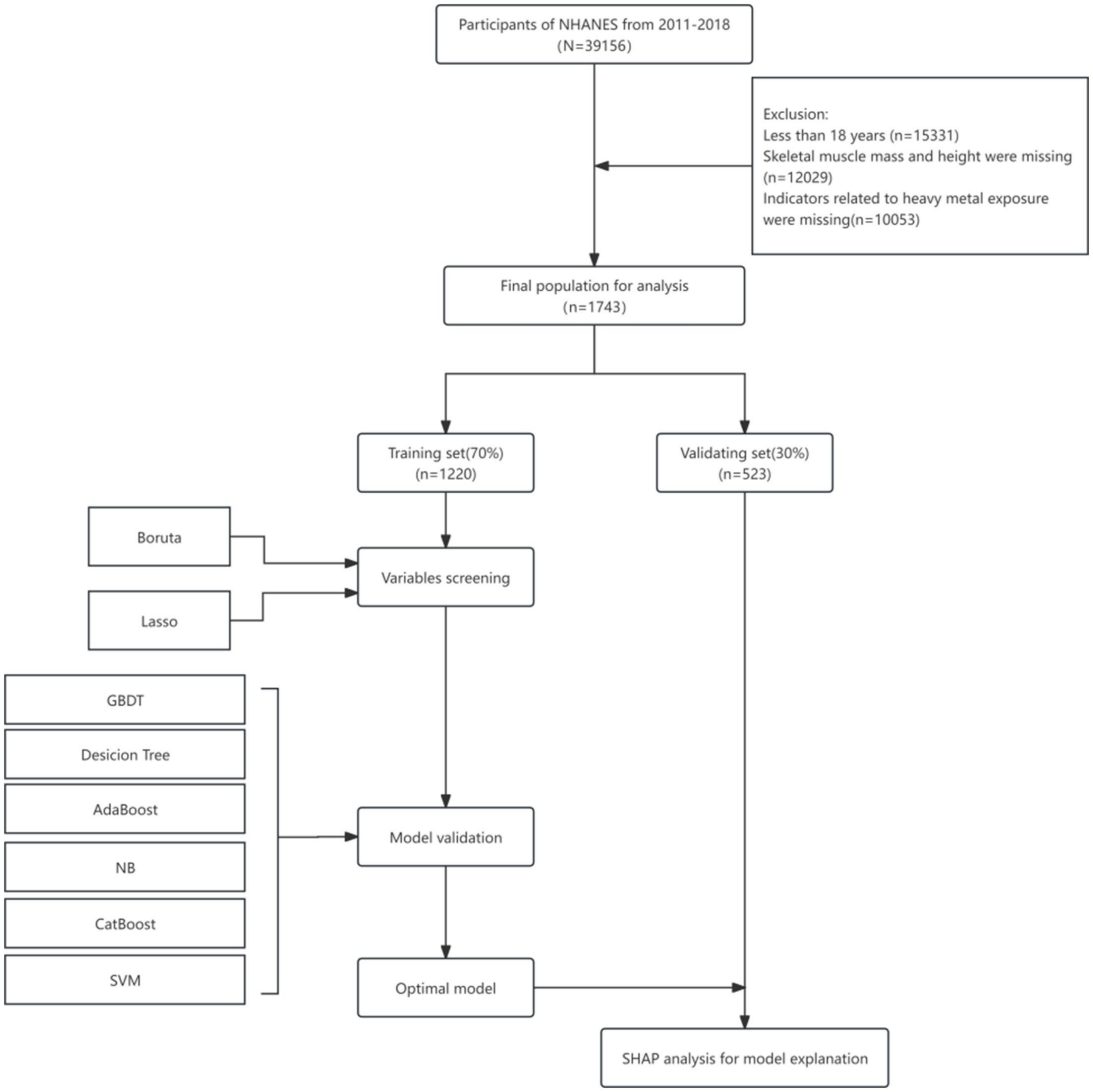
Flowchart of the study design.

The National Health and Nutrition Examination Survey (NHANES) is a program conducted by the National Center for Health Statistics (NCHS), a division of the Centers for Disease Control and Prevention (CDC) in the United States. Its primary objectives are to assess the health and nutritional status of both adults and children, determine the prevalence of major diseases and associated risk factors, and provide data to support the development of nutrition and health policies. The survey received approval from the Research Ethics Review Board of the NCHS, and informed consent was obtained from all participants.^1^ Given the ethical compliance and public accessibility of the database, this study will utilize data from the NHANES database covering the years 2011 to 2018. For the data screening process (see Figure 1), samples with missing indicators related to sarcopenia (including skeletal muscle measurements of the limbs and height), unmeasured indicators associated with heavy metal exposure, and missing values for the remaining covariates exceeding 30% were excluded. Multiple imputation was employed to address the missing data. Ultimately, 3,741 adults (aged ≥ 18 years) with measured levels of nine urinary heavy metals were included in the study.

### 2.2 Assessment of sarcopenia

The appendicular skeletal muscle mass index (ASMI) is a crucial metric for assessing sarcopenia. It is measured using dual-energy X-ray absorptiometry (DXA) and is calculated by incorporating factors such as gender, height, and weight. The index is determined by dividing skeletal muscle mass by the square of height (in meters) (4). It is generally accepted that men with an Appendicular Skeletal Muscle Index (ASMI) of less than 7.0 kg/m^2^ and women with an ASMI of less than 5.4 kg/m^2^ exhibit sarcopenia or may be at risk for developing sarcopenia. For the sake of model stability and sample size considerations, categorizing sarcopenia and potential sarcopenia as two distinct variables when analyzing sarcopenia data will significantly increase the complexity of model evaluation and introduce additional uncertainty. The following aspects may present challenges: (i) Sample Sparsity Problem: The sample size of each subgroup after segmentation may fall below the robust training threshold for machine learning models (e.g., Random Forest/XGBoost). According to the theory of sparse data proposed (26), models are prone to overfitting when the sample size of certain classes is less than 5–10 times the number of features. (ii) SHAP Interpretive Distortion: In cases of unbalanced sample distribution (e.g., *n* < 50 in the confirmed diagnosis group), the game theory-based calculation of SHAP values may exhibit a widening of the confidence interval (27). Consequently, the ranking of feature importance may be significantly altered due to sample perturbation. (iii) Multiple Hypothesis Testing Problem: The introduction of new classification dimensions can compound the variance of model assessment metrics (e.g., AUROC, sensitivity), necessitating data calibration. This, in turn, may lead to a further reduction in statistical efficacy. To address this issue, this paper categorizes both sarcopenia and phenomena that may exhibit sarcopenia symptoms under a single category of sarcopenia. This approach eliminates the previous distinction between the two, ensuring an adequate sample size and enhancing the robustness of the model while minimizing bias and errors that may arise from improper sample segmentation.

### 2.3 Assessment of heavy metal exposure

Inductively coupled plasma mass spectrometry (ICP-MS) is a multi-elemental analytical technique employed to measure various elements in urine, including barium (BA), cadmium (CD), cobalt (CO), cesium (CS), manganese (MN), molybdenum (MO), lead (PB), antimony (SB), tin (SN), thallium (TL), and tungsten (W). Samples were collected using standards from the NHANES database, and creatinine levels were analyzed using the Jaffé reaction, in which creatinine reacts with picric acid in an alkaline solution to form a red creatinine-picric acid complex. The ICP-MS was calibrated using a certified standard, and quality control (QC) procedures included the use of internal standards and duplicate analyses to ensure analytical accuracy and precision. Values below the limit of detection (LOD) were replaced with the square root of the LOD divided by 2. All metal concentrations were adjusted for urinary creatinine (μg/g). For detailed information on urine sampling, storage, measurement, and quality control (QC) procedures for metals, please refer to the NHANES website. Although the concentration of heavy metals in urine primarily indicates recent exposure, it may also indirectly reflect changes in an individual’s short-term health status. In this study, we presented the urinary levels of 11 heavy metal substances: BA, CD, CO, CS, MN, MO, PB, SB, SN, TL, and W, and processed the data in quartiles.

### 2.4 Covariates

In this study, the baseline regression model incorporated demographic characteristics and health factors as covariates.

#### 2.4.1 Demographic characteristics

Four indicators of demographic characteristics—namely age, sex (male/female), ethnicity, and education—were utilized as covariates. Age was analyzed as a continuous variable using actual values, while the other three indicators were coded as categorical variables for numerical analysis. For the gender variable, males were assigned a value of 1, and females were assigned a value of 2. Ethnicity was categorized with values ranging from 1 to 5 for Mexican American, Other Hispanic, Non-Hispanic White, Non-Hispanic Black, and Other Race (including multiracial). Educational attainment was classified on a scale from 1 to 5, corresponding to the following categories: Less than 9th grade, 9th-11th grade (including 12th grade without a diploma), High school graduate/GED or equivalent, some college or AA degree, and College graduate or above.

#### 2.4.2 Health factors

Health factors were specifically evaluated in terms of Life’s Essential 8 (Le8) score and the prevalence of chronic diseases. The Le8 is the most recent measure of cardiovascular health (CVH) developed by the American Heart Association (28). This score is divided into an overall score and individual component scores. The individual score indicators consist of four health behaviors (diet, physical activity, tobacco exposure, and sleep) and four health factors (Body Mass Index (BMI), non-high-density lipoprotein cholesterol (Non-HDL-C) levels, blood glucose, and blood pressure) (29). Based on these scores, a 0–3 coding system was applied at three levels: 0–49 (low level), 50–79 (moderate level), and 80–100 (high level) (30).

Chronic disease conditions were coded based on the presence or absence of 14 specific chronic diseases. These diseases included Hypertension, diabetes, asthma, overweight, arthritis, heart failure, coronary heart disease, angina pectoris, heart attack, stroke, emphysema, chronic bronchitis, liver condition, and cancer. A value of 0 is assigned to indicate the absence of disease, a value of 1 is assigned to indicate the presence of any one of the aforementioned diseases, and a value of 2 is assigned to indicate comorbidities (i.e., the presence of two or more diseases).

### 2.5 Statistical analyses

#### 2.5.1 Baseline regression analysis and logistic regression analysis

In the baseline analysis, continuous variables among the covariates were characterized using medians and interquartile ranges (Q1 and Q3), while categorical variables were presented as the number of cases (denoted as N) along with their corresponding percentages (%). The Wilcoxon rank-sum test and the Pearson chi-square test were employed to compare the differences between the two groups, those with and without oligomyelitis. Subsequently, a logistic regression model was introduced to explore the association between heavy metal exposure and sarcopenia in greater depth, utilizing the odds ratio (OR) and its range within the 95% confidence interval (95% CI). Additionally, adjusted models were constructed by incorporating various categories of covariates. In this context, Model 1 served as the initial model without any adjustments. Considering that the potential confounders addressed in this study encompass two key dimensions—demographic characteristics and health factors—adjustment was implemented in Model 2, which focused on covariates related to demographic characteristics. Furthermore, Model 3 builds upon Model 2 by incorporating adjustments for health factors.

#### 2.5.2 Model development

The dataset comprises a training set of 70% (*n* = 1,220) and a test set of 30% (*n* = 523). The training set is designed to utilize 70% of the data samples to identify latent patterns and intrinsic correlations among the samples, while continuously optimizing the model parameters through iterative methods to validate the model and mitigate the risk of overfitting. The remaining 30% of the data samples are allocated to the test set, which is essential for effectively assessing the model’s generalization capability. Given the complexity of multiple factors influenced by the machine learning (ML) algorithm, the performance of the ML algorithm may exhibit varying degrees of fluctuations and challenges. Consequently, this study employs a combination of Boruta regression and Lasso regression to identify the truly significant features from the provided feature set. Boruta algorithm is a feature selection method based on the Random Forest technique. It primarily filters the feature set by assessing the correlation with the dependent variable through the average reduction in precision values. Additionally, it effectively identifies important variables through multiple dynamic iterations (31). It possesses a robust feature selection capability and can identify the features that are truly valuable for model prediction. The Lasso algorithm is an extension of linear regression that reduces model complexity, enhances the model’s generalization ability, and aids in identifying important predictor variables by incorporating L1 regularization. It effectively maintains strong feature selection performance even when confronted with datasets containing a large number of features. The combination of the two algorithms effectively explores the key features hidden within the data while efficiently eliminating irrelevant or redundant information. It accurately identifies the truly critical influential features from a large set of voluminous data. Based on the specific screened samples of sarcopenia and non-sarcopenia data, we found that the number of non-sarcopenia samples was 1,508, while the number of sarcopenia samples was 235, resulting in an imbalance ratio of 6.417. Due to the imbalance between the samples of sarcopenia and non-sarcopenia data after screening, the study employs a combination of Synthetic Minority Over-sampling Technique (SMOTE) and under-sampling techniques to process the data. This approach ensures a balanced representation of sarcopenia and non-sarcopenia data during model construction. Consequently, the data were balanced between the sarcopenia and non-sarcopenia groups in the model development.

On this basis, six models were employed to model the core variables and assess the risk prediction ability of heavy metal exposure on sarcopenia. The models included Categorical Boosting (CatBoost), Gradient Boosting Decision Tree (Decision Tree, GBDT), Logistic Regression, Random Forest (RF), and Light Gradient Boosting Machine (LGBM). The evaluation metrics used to assess the performance of these models included Area Under the Receiver Operating Characteristic Curve (AUROC), accuracy, balanced accuracy, F1 score, and Matthews Correlation Coefficient (MCC). The initial six machine learning models were evaluated collectively, and the optimal model was selected based on these metrics. The values of AUROC, Accuracy, Balanced Accuracy, and F1 Score range from 0 to 1 (0 to 100%), while the value of the Matthews Correlation Coefficient (MCC) ranges from −1 to 1. The closer the values of all evaluation metrics are to 1, the better the model’s performance. After selecting the optimal model, the SHAP algorithm was employed for interpretability and importance analysis. This method quantifies the contribution of each feature to the model’s output by calculating the Shapley value for each feature. Additionally, the contributions of different feature values to the model output are visually represented by visualizing the SHAP values. After constructing the model, the model-building and evaluation process is repeated using the K-fold cross-validation method. In this approach, each of the K sections is used as a test set in turn, and the average performance is then calculated (32).

All statistical analyses in this study were performed in R software 4.4.1 and Python 3.11 environments. Similarly, a two-tailed test was performed and a *p*-value < 0.05 was considered statistically significant.

## 3 Results

### 3.1 Baseline characteristics and logistic regression

A total of 235 individuals with sarcopenia were selected for the baseline analysis and compared with 1,508 individuals without sarcopenia. The baseline characteristics are presented in Table 1. Among the 1,743 participants in this study (mean age: 37.00 years), 295 (16.92%) identified as Mexican-American, 189 (10.84%) as other Hispanic, 586 (33.62%) as non-Hispanic White, 353 (20.25%) as non-Hispanic Black, and 320 (18.36%) as Other Race or Multiracial. There was a significant difference in racial distribution between the two groups (*p* < 0.001). Starting with the health factor covariates, we observed significant differences between the two samples regarding chronic diseases, including hypertension, diabetes, overweight, heart disease, emphysema, and sarcopenia (*p* < 0.05). Notably, hypertension and overweight were significantly more prevalent (*p* < 0.001). Additionally, the two groups exhibited significant differences in le8, le8 pa, le8 BMI, le8 non hdl, le8 glucose, and le8 bp 6 indicators were significantly different (*p* < 0.05), and le8, le8 BMI, le8 non hdl, and le8 glucose demonstrated even greater significance (*p* < 0.001). Among the indicators related to heavy metal exposure, we found that the differences were not statistically significant only for MN and PB (*p* > 0.05). In contrast, the other heavy metal exposures showed significant differences (*p* < 0.05), with notably higher differences between the two groups observed for CS, SB, SN, and TL (*p* < 0.001).

**Table 1:** Comparison of baseline characteristics between sarcopenia and non-sarcopenia groups.

Variable	Non-sarcopenia	Sarcopenia	*p*-value
	*N* = 1,508	*N* = 235	
Age (years), Median (IQR)	37.20 (12.60)	35.72 (13.58)	0.117
Sex, *N* (%)			0.939
Male	766.00 (50.80)	120.00 (51.06)	
Female	742.00 (49.20)	115.00 (48.94)	
Race, *N* (%)			**<0.001**
Mexican American	266.00 (17.64)	29.00 (12.34)	
Other Hispanics	171.00 (11.34)	18.00 (7.66)	
Non-Hispanic white	505.00 (33.49)	81.00 (34.47)	
Non-Hispanic blacks	340.00 (22.55)	13.00 (5.53)	
Other (including multiracial)	226.00 (14.99)	94.00 (40.00)	
Educational level, *N* (%)			0.196
Below 9th grade	82.00 (5.44)	12.00 (5.11)	
Grades 9–11 (including those without a grade 12 diploma)	195.00 (12.93)	40.00 (17.02)	
High school graduation or equivalent	382.00 (25.33)	49.00 (20.85)	
Some college or AA degree	452.00 (29.97)	63.00 (26.81)	
Bachelor’s degree or higher	397.00 (26.33)	71.00 (30.21)	
Hypertension, *N* (%)			**<0.001**
No	1,154.00 (76.53)	204.00 (86.81)	
Yes	354.00 (23.47)	31.00 (13.19)	
Diabetes, *N* (%)			**0.004**
No	1,393.00 (92.37)	229.00 (97.45)	
Yes	115.00 (7.63)	6.00 (2.55)	
Asthma, *N* (%)			0.077
No	1,260.00 (83.55)	207.00 (88.09)	
Yes	248.00 (16.45)	28.00 (11.91)	
Overweight, *N* (%)			**<0.001**
No	937.00 (62.14)	226.00 (96.17)	
Yes	571.00 (37.86)	9.00 (3.83)	
Arthritis, *N* (%)			0.326
No	1,327.00 (88.00)	212.00 (90.21)	
Yes	181.00 (12.00)	23.00 (9.79)	
Heart Failure, *N* (%)			0.613
No	1,494.00 (99.07)	232.00 (98.72)	
Yes	14.00 (0.93)	3.00 (1.28)	
Coronary heart disease, *N* (%)			0.163
No	1,492.00 (98.94)	230.00 (97.87)	
Yes	16.00 (1.06)	5.00 (2.13)	
Angina pectoris, *N* (%)			0.275
No	1,494.00 (99.07)	231.00 (98.30)	
Yes	14.00 (0.93)	4.00 (1.70)	
Heart attack, *N* (%)			**0.003**
No	1,496.00 (99.20)	228.00 (97.02)	
Yes	12.00 (0.80)	7.00 (2.98)	
Stroke, *N* (%)			0.775
No	1,486.00 (98.54)	231.00 (98.30)	
Yes	22.00 (1.46)	4.00 (1.70)	
Emphysema, *N* (%)			**0.043**
No	1,500.00 (99.47)	231.00 (98.30)	
Yes	8.00 (0.53)	4.00 (1.70)	
Chronic Bronchitis, *N* (%)			0.389
No	1,449.00 (96.09)	223.00 (94.89)	
Yes	59.00 (3.91)	12.00 (5.11)	
Liver Condition, *N* (%)			0.570
No	1,459.00 (96.75)	229.00 (97.45)	
Yes	49.00 (3.25)	6.00 (2.55)	
Cancer, *N* (%)			0.858
No	1,460.00 (96.82)	227.00 (96.60)	
Yes	48.00 (3.18)	8.00 (3.40)	
le8, *N* (%)			**<0.001**
49 ≥ x ≥ 0	118.00 (7.82)	7.00 (2.98)	
79 ≥ x ≥ 50	827.00 (54.84)	104.00 (44.26)	
100 ≥ x ≥ 80	563.00 (37.33)	124.00 (52.77)	
le8 hei, *N* (%)			0.974
49 ≥ x ≥ 0	820.00 (54.38)	129.00 (54.89)	
79 ≥ x ≥ 50	428.00 (28.38)	65.00 (27.66)	
100 ≥ x ≥ 80	260.00 (17.24)	41.00 (17.45)	
le8 pa, *N* (%)			**0.009**
49 ≥ x ≥ 0	367.00 (24.34)	79.00 (33.62)	
79 ≥ x ≥ 50	52.00 (3.45)	6.00 (2.55)	
100 ≥ x ≥ 80	1,089.00 (72.21)	150.00 (63.83)	
le8 smoke, *N* (%)			0.506
49 ≥ x ≥ 0	263.00 (17.44)	43.00 (18.30)	
79 ≥ x ≥ 50	54.00 (3.58)	5.00 (2.13)	
100 ≥ x ≥ 80	1,191.00 (78.98)	187.00 (79.57)	
le8 sleep, *N* (%)			0.713
49 ≥ x ≥ 0	267.00 (17.71)	44.00 (18.72)	
79 ≥ x ≥ 50	330.00 (21.88)	46.00 (19.57)	
100 ≥ x ≥ 80	911.00 (60.41)	145.00 (61.70)	
le8 BMI, *N* (%)			**<0.001**
49 ≥ x ≥ 0	479.00 (31.76)	1.00 (0.43)	
79 ≥ x ≥ 50	470.00 (31.17)	27.00 (11.49)	
100 ≥ x ≥ 80	559.00 (37.07)	207.00 (88.09)	
le8 non hdl, *N* (%)			**<0.001**
49 ≥ x ≥ 0	501.00 (33.22)	54.00 (22.98)	
79 ≥ x ≥ 50	362.00 (24.01)	50.00 (21.28)	
100 ≥ x ≥ 80	645.00 (42.77)	131.00 (55.74)	
le8 glucose, *N* (%)			**<0.001**
49 ≥ x ≥ 0	103.00 (6.83)	4.00 (1.70)	
79 ≥ x ≥ 50	171.00 (11.34)	12.00 (5.11)	
100 ≥ x ≥ 80	1,234.00 (81.83)	219.00 (93.19)	
le8 bp, *N* (%)			**0.022**
49 ≥ x ≥ 0	164.00 (10.88)	14.00 (5.96)	
79 ≥ x ≥ 50	387.00 (25.66)	53.00 (22.55)	
100 ≥ x ≥ 80	957.00 (63.46)	168.00 (71.49)	
BA, Median (IQR)			**0.004**
Q1	359.00 (23.81)	81.00 (34.47)	
Q2	381.00 (25.27)	54.00 (22.98)	
Q3	379.00 (25.13)	54.00 (22.98)	
Q4	389.00 (25.80)	46.00 (19.57)	
CD, Median (IQR)			**0.026**
Q1	376.00 (24.93)	62.00 (26.38)	
Q2	377.00 (25.00)	68.00 (28.94)	
Q3	386.00 (25.60)	39.00 (16.60)	
Q4	369.00 (24.47)	66.00 (28.09)	
CO, Median (IQR)			**0.011**
Q1	362.00 (24.01)	75.00 (31.91)	
Q2	378.00 (25.07)	66.00 (28.09)	
Q3	390.00 (25.86)	44.00 (18.72)	
Q4	378.00 (25.07)	50.00 (21.28)	
CS, Median (IQR)			**<0.001**
Q1	354.00 (23.47)	87.00 (37.02)	
Q2	369.00 (24.47)	68.00 (28.94)	
Q3	387.00 (25.66)	43.00 (18.30)	
Q4	398.00 (26.39)	37.00 (15.74)	
MN, Median (IQR)			0.414
Q1	141.00 (9.35)	19.00 (8.09)	
Q2	800.00 (53.05)	138.00 (58.72)	
Q3	209.00 (13.86)	31.00 (13.19)	
Q4	358.00 (23.74)	47.00 (20.00)	
MO, Median (IQR)			**0.002**
Q1	359.00 (23.81)	79.00 (33.62)	
Q2	373.00 (24.73)	61.00 (25.96)	
Q3	382.00 (25.33)	54.00 (22.98)	
Q4	394.00 (26.13)	41.00 (17.45)	
PB, Median (IQR)			0.080
Q1	377.00 (25.00)	73.00 (31.06)	
Q2	377.00 (25.00)	55.00 (23.40)	
Q3	369.00 (24.47)	62.00 (26.38)	
Q4	385.00 (25.53)	45.00 (19.15)	
SB, Median (IQR)			**<0.001**
Q1	562.00 (37.27)	123.00 (52.34)	
Q2	301.00 (19.96)	37.00 (15.74)	
Q3	288.00 (19.10)	32.00 (13.62)	
Q4	357.00 (23.67)	43.00 (18.30)	
SN, Median (IQR)			**<0.001**
Q1	348.00 (23.08)	88.00 (37.45)	
Q2	383.00 (25.40)	61.00 (25.96)	
Q3	380.00 (25.20)	51.00 (21.70)	
Q4	397.00 (26.33)	35.00 (14.89)	
TL, Median (IQR)			**<0.001**
Q1	385.00 (25.53)	99.00 (42.13)	
Q2	353.00 (23.41)	61.00 (25.96)	
Q3	401.00 (26.59)	41.00 (17.45)	
Q4	369.00 (24.47)	34.00 (14.47)	
W, Median (IQR)			**0.026**
Q1	437.00 (28.98)	91.00 (38.72)	
Q2	361.00 (23.94)	49.00 (20.85)	
Q3	355.00 (23.54)	49.00 (20.85)	
Q4	355.00 (23.54)	46.00 (19.57)	

Median (IQR) or Frequency (%). Bold values indicate statistically significant differences between groups (*p* < 0.05).

**Figure 2:**
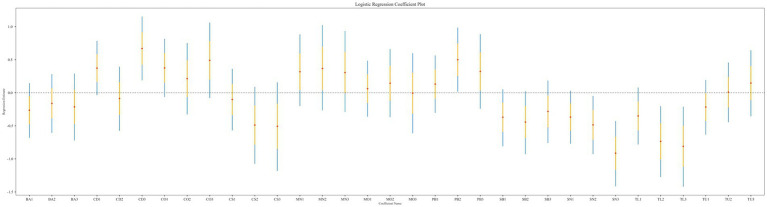
Forest plot of logistic regression results showing odds ratios for sarcopenia across quartiles of metal exposure.

The results of the logistic regression analysis indicated a correlation between heavy metal exposure and sarcopenia. As shown in Figure 2, CD, PB, SN, and TL were significantly associated with the risk of sarcopenia. The logistic regression analysis results (see Table 2) revealed that, in Model 1, which did not adjust for covariates, the risk of sarcopenia was significantly higher for CD (Q3: OR = 1.953, 95% CI = 1.208–3.172, *p* = 0.006), PB (Q2: OR = 1.649, 95% CI = 1.018–2.678, *p* = 0.042), SN (Q2: OR = 0.616, 95% CI = 0.395–0.953, *p* = 0.031; Q3: OR = 0.401, 95% CI = 0.243–0.651, *p* = 0.000), and TL (Q2: OR = 0.480, 95% CI = 0.280–0.817, *p* = 0.007; Q3: OR = 0.445, 95% CI = 0.241–0.811, *p* = 0.009). Among the substances studied, CD exposure must reach a concentration level of Q3 to exhibit toxic effects. The CD muscle toxin may disrupt calcium metabolism or mitochondrial function; however, it requires a Q3 concentration to surpass the physiological compensatory mechanisms. The results for PB were significant in Q2 but not in Q3 or Q4, which may have been influenced by confounding factors, or the accuracy of the assay may have diminished at higher concentrations of PB. This necessitates further adjustments to analyze the covariates. Both SN and TL demonstrated a reduced risk (OR < 1) in Q2 and Q3, with the magnitude of the effect increasing with exposure. In Q3, both substances continued to show a reduced risk (OR < 1), and effect sizes also increased with exposure.

**Table 2:** Correlation analysis between heavy metal exposure and sarcopenia.

Variable name	Model 1		Model 2		Model 3	
	OR (95%CI)	*p-*value	OR (95% CI)	*p*-value	OR (95% CI)	*p*-value
BA						
Q2	0.768 (0.507–1.158)	0.210	0.593 (0.382–0.915)	0.019	0.603 (0.366–0.986)	0.045
Q3	0.852 (0.545–1.325)	0.479	0.636 (0.394–1.019)	0.061	0.651 (0.376–1.120)	0.123
Q4	0.808 (0.486–1.337)	0.409	0.538 (0.311–0.924)	0.025	0.593 (0.317–1.101)	0.099
CD						
Q2	1.452 (0.965–2.191)	0.074	1.512 (0.972–2.358)	0.067	1.488 (0.915–2.428)	0.110
Q3	0.917 (0.563–1.484)	0.726	1.025 (0.595–1.760)	0.927	0.971 (0.536–1.753)	0.922
Q4	1.953 (1.208–3.172)	0.006	2.396 (1.333–4.339)	0.004	1.838 (0.938–3.622)	0.077
CO						
Q2	1.456 (0.938–2.264)	0.094	1.550 (0.976–2.467)	0.064	1.823 (1.078–3.098)	0.026
Q3	1.238 (0.721–2.121)	0.437	1.306 (0.735–2.318)	0.362	1.729 (0.906–3.310)	0.097
Q4	1.635 (0.924–2.892)	0.091	1.784 (0.955–3.340)	0.070	2.044 (1.011–4.150)	0.047
CS						
Q2	0.903 (0.567–1.437)	0.667	0.910 (0.559–1.481)	0.705	0.889 (0.514–1.536)	0.674
Q3	0.614 (0.342–1.095)	0.100	0.569 (0.308–1.042)	0.069	0.544 (0.274–1.071)	0.080
Q4	0.602 (0.307–1.170)	0.137	0.625 (0.309–1.254)	0.188	0.745 (0.337–1.635)	0.464
MN						
Q2	1.375 (0.818–2.420)	0.247	1.561 (0.904–2.812)	0.122	1.411 (0.761–2.715)	0.287
Q3	1.445 (0.767–2.784)	0.261	1.467 (0.755–2.911)	0.263	1.405 (0.665–3.021)	0.378
Q4	1.358 (0.748–2.546)	0.325	1.446 (0.777–2.776)	0.255	1.406 (0.701–2.899)	0.345
MO						
Q2	1.064 (0.696–1.625)	0.773	1.037 (0.665–1.616)	0.873	0.976 (0.597–1.596)	0.924
Q3	1.156 (0.691–1.935)	0.581	1.056 (0.617–1.809)	0.842	1.255 (0.685–2.307)	0.463
Q4	0.994 (0.542–1.821)	0.986	0.954 (0.508–1.787)	0.882	0.931 (0.472–1.837)	0.837
PB						
Q2	1.140 (0.738–1.759)	0.552	1.175 (0.746–1.849)	0.486	0.972 (0.590–1.598)	0.912
Q3	1.649 (1.018–2.678)	0.042	1.815 (1.083–3.053)	0.024	1.301 (0.732–2.316)	0.370
Q4	1.383 (0.786–2.427)	0.259	1.613 (0.880–2.951)	0.121	1.001 (0.502–1.984)	0.998
SB						
Q2	0.691 (0.445–1.055)	0.092	0.824 (0.522–1.282)	0.398	0.979 (0.594–1.595)	0.934
Q3	0.642 (0.396–1.023)	0.067	0.861 (0.516–1.415)	0.561	1.170 (0.658–2.058)	0.588
Q4	0.755 (0.469–1.203)	0.241	0.952 (0.575–1.565)	0.848	1.125 (0.642–1.961)	0.679
SN						
Q2	0.693 (0.463–1.031)	0.072	0.831 (0.547–1.256)	0.380	0.917 (0.579–1.448)	0.710
Q3	0.616 (0.395–0.953)	0.031	0.838 (0.526–1.327)	0.452	0.795 (0.469–1.339)	0.390
Q4	0.401 (0.243–0.651)	0.000	0.519 (0.309–0.858)	0.012	0.589 (0.329–1.041)	0.071
TL						
Q2	0.705 (0.458–1.082)	0.111	0.689 (0.437–1.081)	0.106	0.716 (0.426–1.198)	0.205
Q3	0.480 (0.280–0.817)	0.007	0.437 (0.248–0.765)	0.004	0.428 (0.224–0.809)	0.009
Q4	0.445 (0.241–0.811)	0.009	0.335 (0.175–0.630)	0.001	0.369 (0.177–0.757)	0.007
W						
Q2	0.807 (0.531–1.214)	0.307	0.800 (0.517–1.227)	0.310	0.919 (0.561–1.496)	0.735
Q3	1.009 (0.641–1.580)	0.967	1.012 (0.628–1.623)	0.962	0.940 (0.553–1.594)	0.820
Q4	1.158 (0.700–1.902)	0.563	1.038 (0.610–1.754)	0.890	1.296 (0.711–2.359)	0.397

OR, odds ratio; CI, confidence interval. Model 1 adjusted no confounding factor; Model 2 adjusted age, sex, race, education level; Model 3 adjusted Le8 scores (le8, le8 BMI, le8 bp, le8 pa, le8 hei, le8 smoke, le8 non hdl, le8 glucose and le8 sleep) and 14 chronic diseases (Hypertensive, Diabetes, Eczema, Overweight, Arthritis, Heart Failure, Coronary heart disease, Angina pectoris, Heart Disease, Stroke, Emphysema, Chronic Bronchitis, Liver Disease and Cancer) under the basic of Model 2.

In Model 2, which adjusted for demographic characteristics, the associations remained significant: CD (Q3: OR = 2.396, 95% CI = 1.333–4.339, *p* = 0.004), PB (Q2: OR = 1.815, 95% CI = 1.083–3.053, *p* = 0.024), SN (Q3: OR = 0.519, 95% CI = 0.309–0.858, *p* = 0.012), and TL (Q2: OR = 0.437, 95% CI = 0.248–0.765, p = 0.004; Q3: OR = 0.335, 95% CI = 0.175–0.630, *p* = 0.001). Additionally, BA (Q1: OR = 0.593, 95% CI = 0.382–0.915, *p* = 0.019; Q3: OR = 0.538, 95% CI = 0.311–0.924, *p* = 0.025) demonstrated a significant correlation after adjusting for demographic characteristics. At this point, the risk effect of CD and PB was heightened, while the protective effect of TL continued to increase. In contrast, the protective effect of SN may have been diminished by age-related factors. Some of this protective effect was reduced after adjustment, likely due to the greater muscle mass observed in the younger group. Additionally, the inclusion of BA demonstrated a protective effect in both quartiles Q1 and Q3 (OR ≈ 0.55).

When health-related covariates were incorporated in Model 3, we found that BA (Q1: OR = 0.603, 95% CI = 0.366–0.986, *p* = 0.045), CO (Q1: OR = 1.823, 95% CI = 1.078–3.098, *p* = 0.026; Q3: OR = 2.044, 95% CI = 1.011–4.150, *p* = 0.047), and TL (Q2: OR = 0.428, 95% CI = 0.224–0.809, *p* = 0.009; Q3: OR = 0.369, 95% CI = 0.177–0.757, *p* = 0.007) were correlated with sarcopenia. In this process, we found that the protective effect of TL remained significant after several adjustments (OR ≈ 0.4), and the confidence interval did not significantly widen. In BA-Q1, the protective effect continued to be significant (OR ≈ 0.6); however, this effect disappeared in Q3. The apparent protectiveness observed in Q3 may be attributed to a healthier lifestyle (e.g., exercise, diet) within this population. The true effect was diminished after adjustments, which warrants further analysis in the discussion. Additionally, the emerging risk factor CO increased the risk in both Q1 and Q3 intervals (OR ≈ 1.8–2.0).

In summary, the logistic regression analysis confirmed that BA, CD, CO, PB, SN, and TL were associated with sarcopenia across multiple models.

### 3.2 Selection of model variables

**Figure 3:**
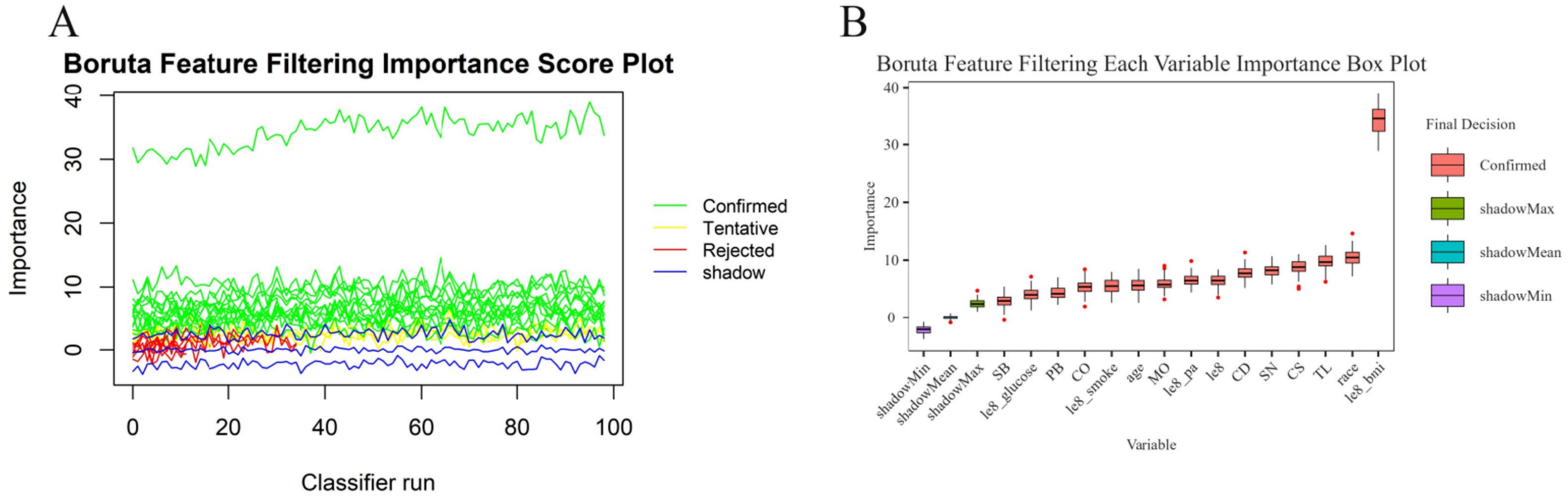
Boruta algorithm feature screening plot. **(A)** Boruta feature screening importance score plot. **(B)** Corresponding importance box plots for each variable of Boruta feature screening.

The study conducted variable screening using a combination of the Boruta and Lasso algorithms. The Boruta algorithm identified 15 variables (see Figure 3). Corresponding to the red module in Figure 3B, the variables identified through the shaded feature variable training include le8 BMI, race, TL, CS, SN, CD, le8, le8 pa, MO, age, le8 smoke, CO, PB, le8 glucose, and SB.

**Figure 4:**
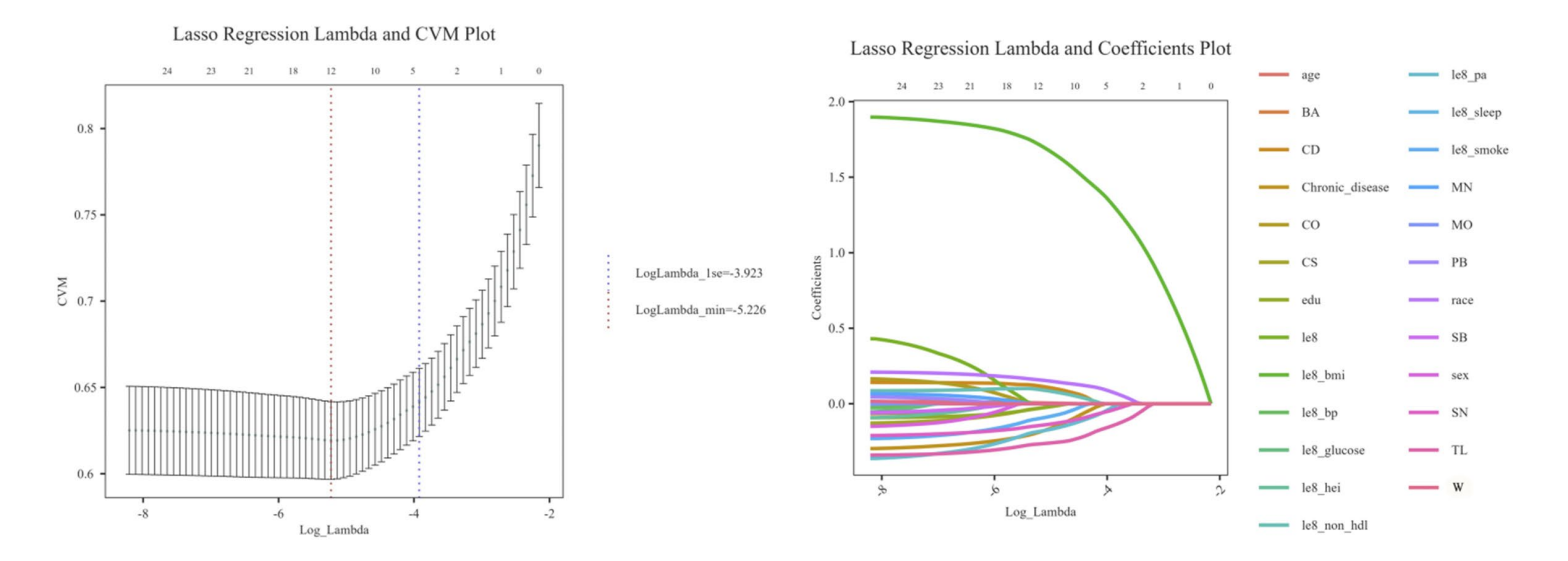
Lasso algorithm eigenvalue screening plot. **(A)** Lasso regression Lambda and CVM plot. **(B)** Lasso regression Lambda and coefficients plot.

Lasso analysis was further conducted by setting the number of Lambda values to 100, the Lambda filtering threshold to minimum, the loss type to deviance, and determining the optimal regularization parameter (*λ*) through 10-fold cross-validation. In Figure 4A, Lambda min (−5.226) represents the value of λ that minimizes the CVM to its minimum value, and Lambda 1se (−3.923) indicates the maximum λ value within one standard error of the minimum CVM. Additionally, as shown in Figure 4B, when Lambda min is −5.226, the variables converge relatively well, resulting in the identification of a total of 12 variables. These variables include age, race, edu, CD, CS, SN, TL, Chronic disease, le8 pa, le8 smoke, le8 BMI, le8 non hdl.

**Figure 5:**
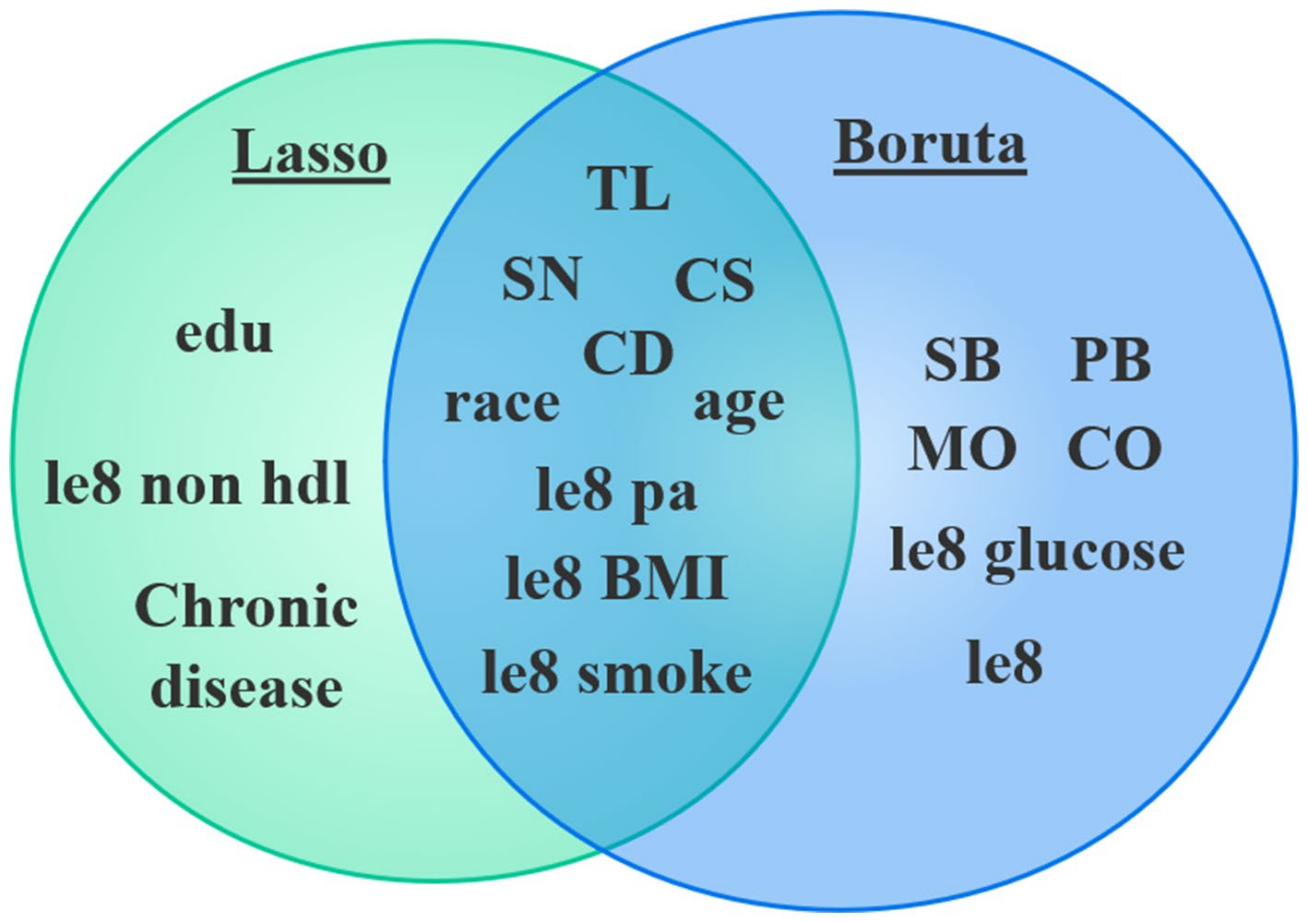
Boruta and Lasso feature selection overlap.

By combining the two algorithms (see Figure 5), we will take the intersection of the identified variables to construct the model, which includes CD, CS, SN, TL, age, race, le8 pa, le8 smoke, and le8 BMI, totaling 9 variables.

### 3.3 Model evaluation and comparison

**Figure 6:**
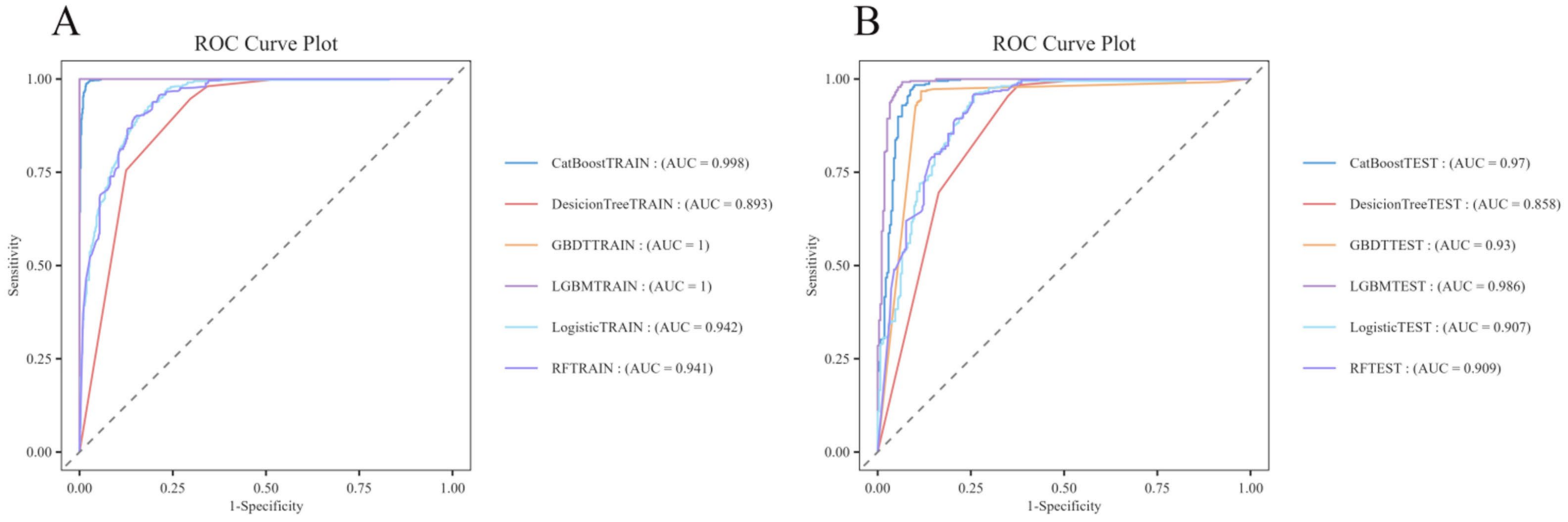
ROC curves of the train and test sets of 6 ML models. **(A)** ROC curves of the train set. **(B)** ROC curves of the test set.

The training set ROC curves for GBDT, RF, Logistic Regression, LGBM, Decision Tree, and CatBoost are presented in Figure 6A, while Figure 6B displays the test set ROC curves. By evaluating both the training and test sets, we determine that the LGBM model demonstrates the highest predictive performance, achieving a training set accuracy of 1.00000, a recall of 1.00000, an F1-score of 1.00000, and an MCC of 1.00000. In comparison, the test set results show an accuracy of 0.96112, a recall of 0.99185, an F1-score of 0.96689, and an MCC of 0.92159, indicating superior performance relative to the other models (see Table 3 for details). Additionally, the AUROC values were 1.000 for the training set and 0.986 for the test set, confirming LGBM as the most suitable model for adaptation. Consequently, LGBM was selected for the construction of the predictive model in the subsequent study.

**Table 3:** Evaluation results of training set and test set.

Model name	Accuracy	Recall	F1-score	MCC
GBDTTRAIN	1.00000	1.00000	1.00000	1.00000
RFTRAIN	0.87392	0.96767	0.89866	0.74727
LogisticTRAIN	0.87392	0.90416	0.89231	0.74069
LGBMTRAIN	1.00000	1.00000	1.00000	1.00000
DecisionTreeTRAIN	0.84390	0.94688	0.87513	0.68395
CatBoostTRAIN	0.98132	0.99654	0.98404	0.96198
GBDTTEST	0.92846	0.96196	0.93899	0.85407
RFTEST	0.85070	0.96196	0.88060	0.70288
LogisticTEST	0.84914	0.91304	0.87386	0.69102
LGBMTEST	0.96112	0.99185	0.96689	0.92159
DecisionTreeTEST	0.82426	0.95380	0.86135	0.64992
CatBoostTEST	0.93624	0.98370	0.94641	0.87180

### 3.4 Optimal model validation

**Figure 7:**
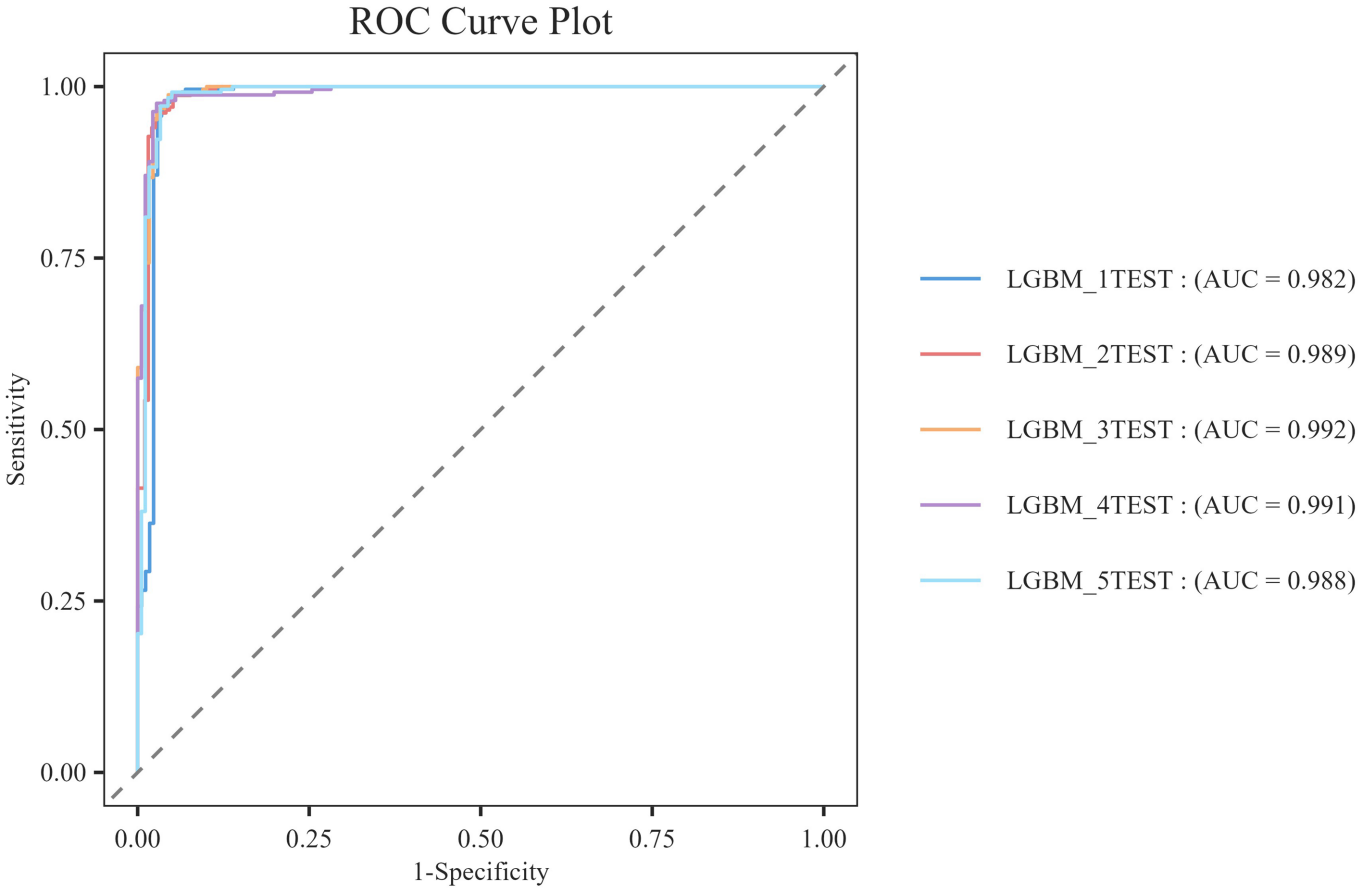
ROC curve for the 5-fold test.

To effectively address the issues of bias and variance during model selection, this study employs 5-fold cross-validation to accurately evaluate the performance of machine learning models. The dataset is divided into five disjoint subsets, with four subsets used as the training set and the remaining subset as the validation set in each iteration. This process is repeated five times to obtain a robust estimate of model performance. The results of the cross-validation (see Figure 7) indicate that the LGBM model demonstrates exceptional predictive performance, achieving an accuracy of 0.9884 ± 0.00684.

**Figure 8:**
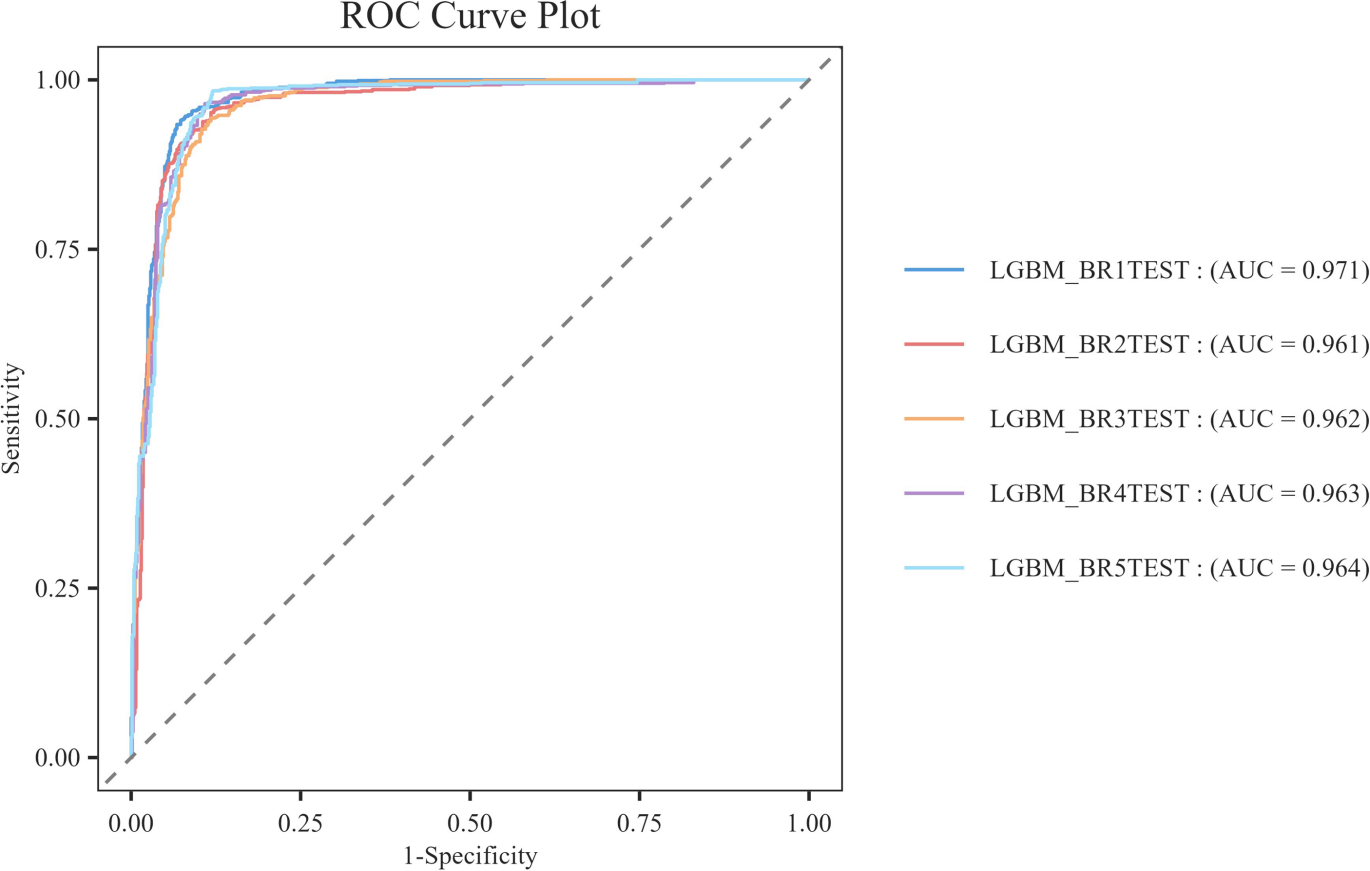
ROC curve for Bootstrap evaluation.

At the same time, resampling statistical validation for Bootstrap error assessment is conducted. The model’s performance is validated multiple times by resampling the training set using Bootstrap techniques to calculate the mean and variance of the performance metrics. This process generates multiple new datasets (referred to as Bootstrap samples) by randomly sampling from the original dataset with replacement, and then trains and evaluates the model on each sample. The Bootstrap method is particularly effective for addressing bias and variance issues that may arise during the model selection process, as it provides a robust estimation of model performance and ensures the validity and robustness of the selected models. According to Figure 8, we can observe that the LGBM_BR1TEST model exhibits the best performance, achieving an AUC value of 0.971, while the other models all have AUC values of 0.961 or higher, indicating good classification performance.

### 3.5 Visual analysis of feature importance

**Figure 9:**
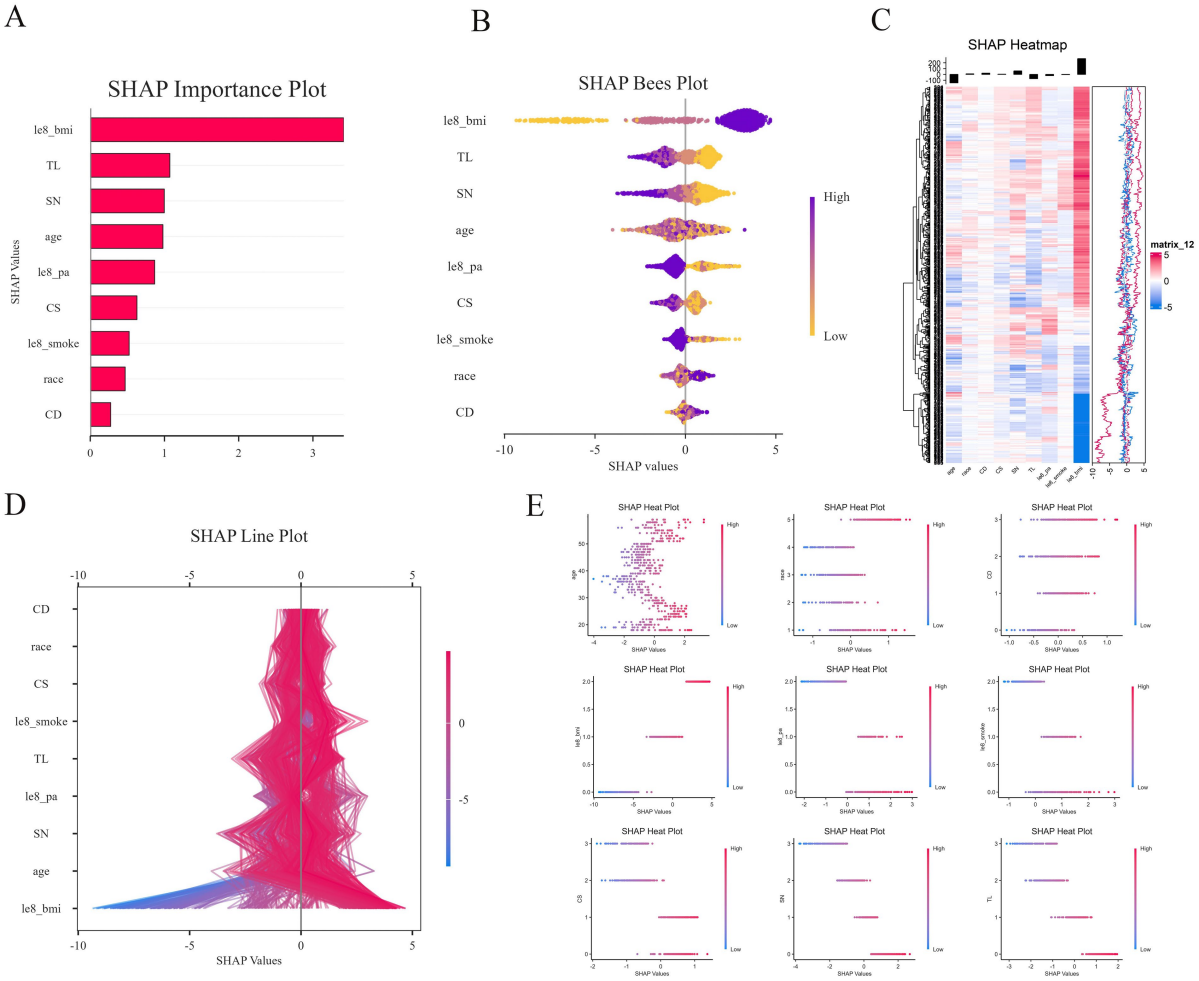
SHAP diagram. **(A)** SHAP importance plot; **(B)** SHAP bees plot; **(C)** SHAP heatmap; **(D)** SHAP line plot; **(E)** SHAP heat plot with age, race, Le8 BMI, Le8 smoke, Le8 pa, CD, CS, SN, and TL.

In this study, SHAP analysis was employed to evaluate the extent to which each input feature in LGBM contributes to the final output, as well as to establish a hierarchy of relative importance. The significant role of le8 BMI in predicting sarcopenia risk is illustrated in Figure 9A. Larger SHAP values indicate a greater influence on the model output, with importance decreasing in descending order from top to bottom. Other features, including TL, SN, age, le8 pa, CS, le8 smoke, race, and CD metrics, also demonstrated substantial predictive power. Conversely, the swarm plot (Figure 9B) displays the distribution of SHAP values for heavy metal exposure and covariates, along with the direction of their influence on the model output. Regarding heavy metal exposure, we found that TL and SN contributed negatively to the prediction of sarcopenia risk. When combined with the scatter plot (Figure 9E), a relatively complex nonlinear relationship emerges for CD, a heavy metal element, in relation to sarcopenia prediction. Among the covariates, le8 BMI exhibited a positive predictive relationship with sarcopenia. Additionally, age displayed a complex linear relationship that made its predictive direction challenging to ascertain, while le8 pa and le8 smoke demonstrated a clear negative predictive relationship. Figure 9D reveals a more intricate relationship between race, a covariate, and sarcopenia, indicating that its role in sarcopenia should be carefully considered to avoid potential bias.

### 3.6 Interaction analysis

**Figure 10:**
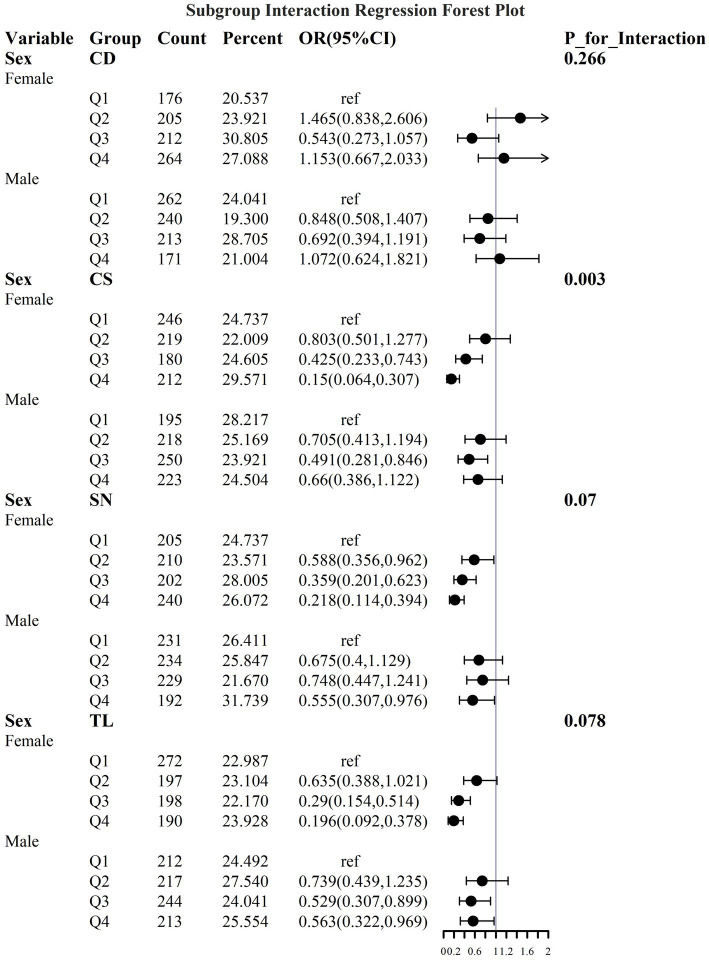
Logistic regression forest plot based on gender differences.

**Figure 11:**
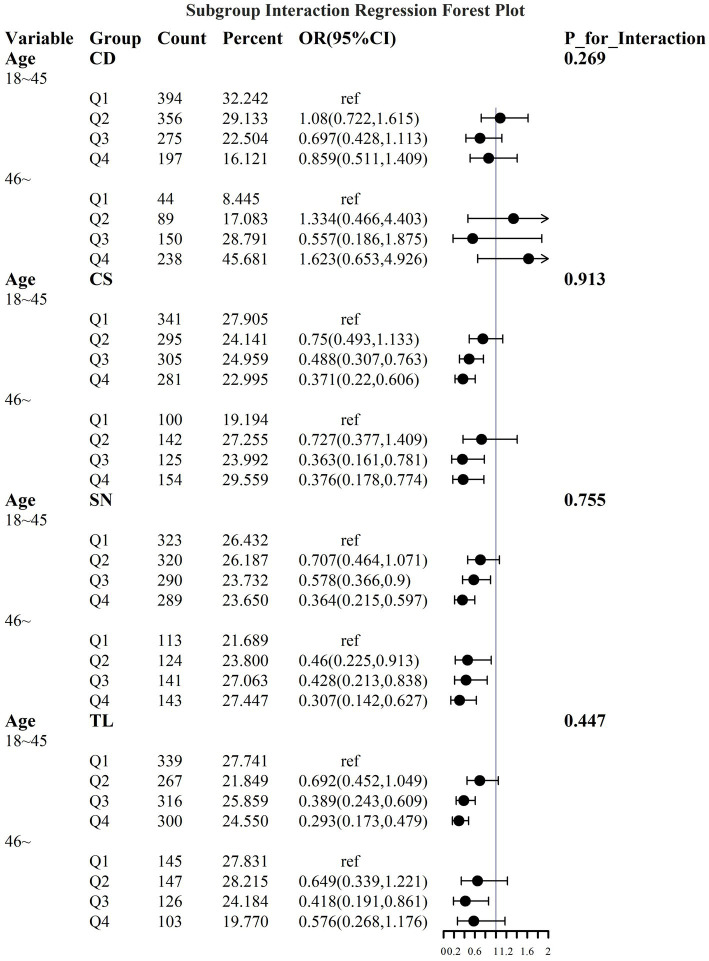
Logistic regression forest plot based on age differences.

**Figure 12:**
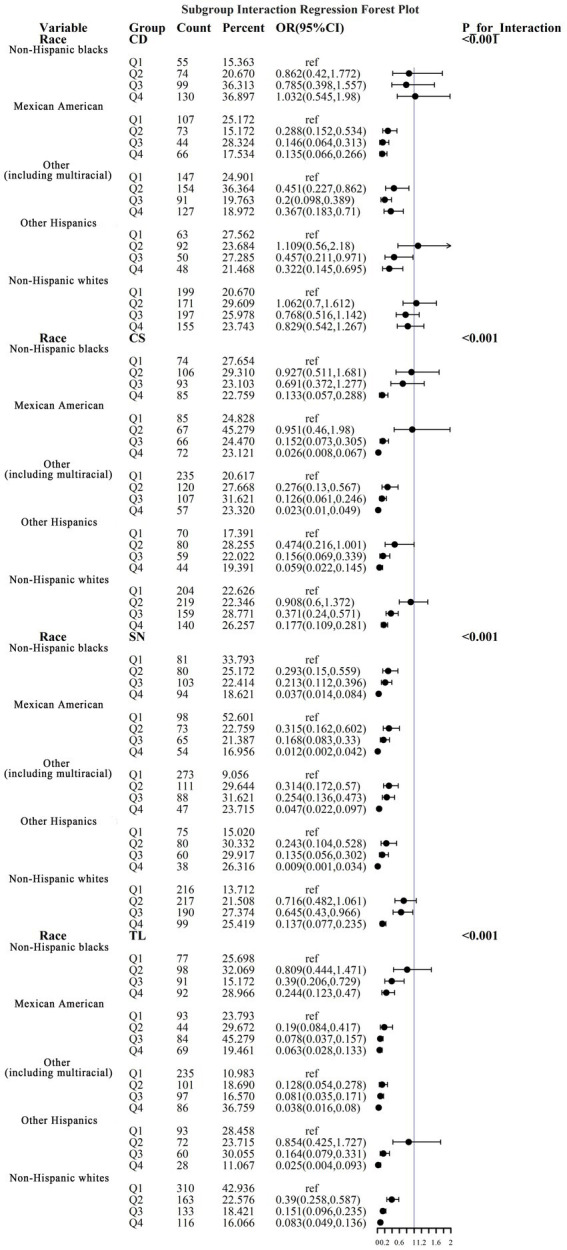
Logistic regression forest plots based on racial differences.

**Figure 13:**
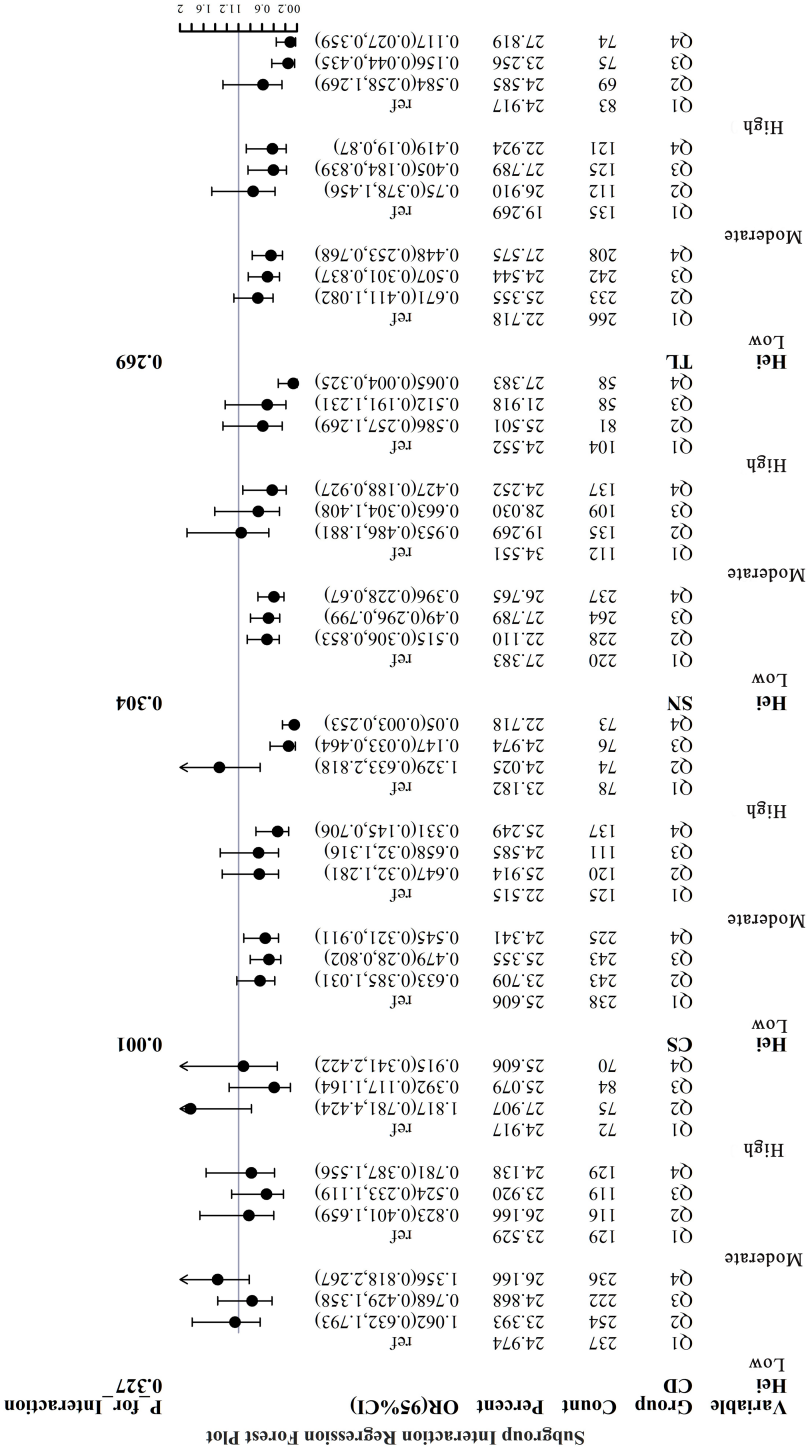
Logistic regression forest plot based on dietary differences.

**Figure 14:**
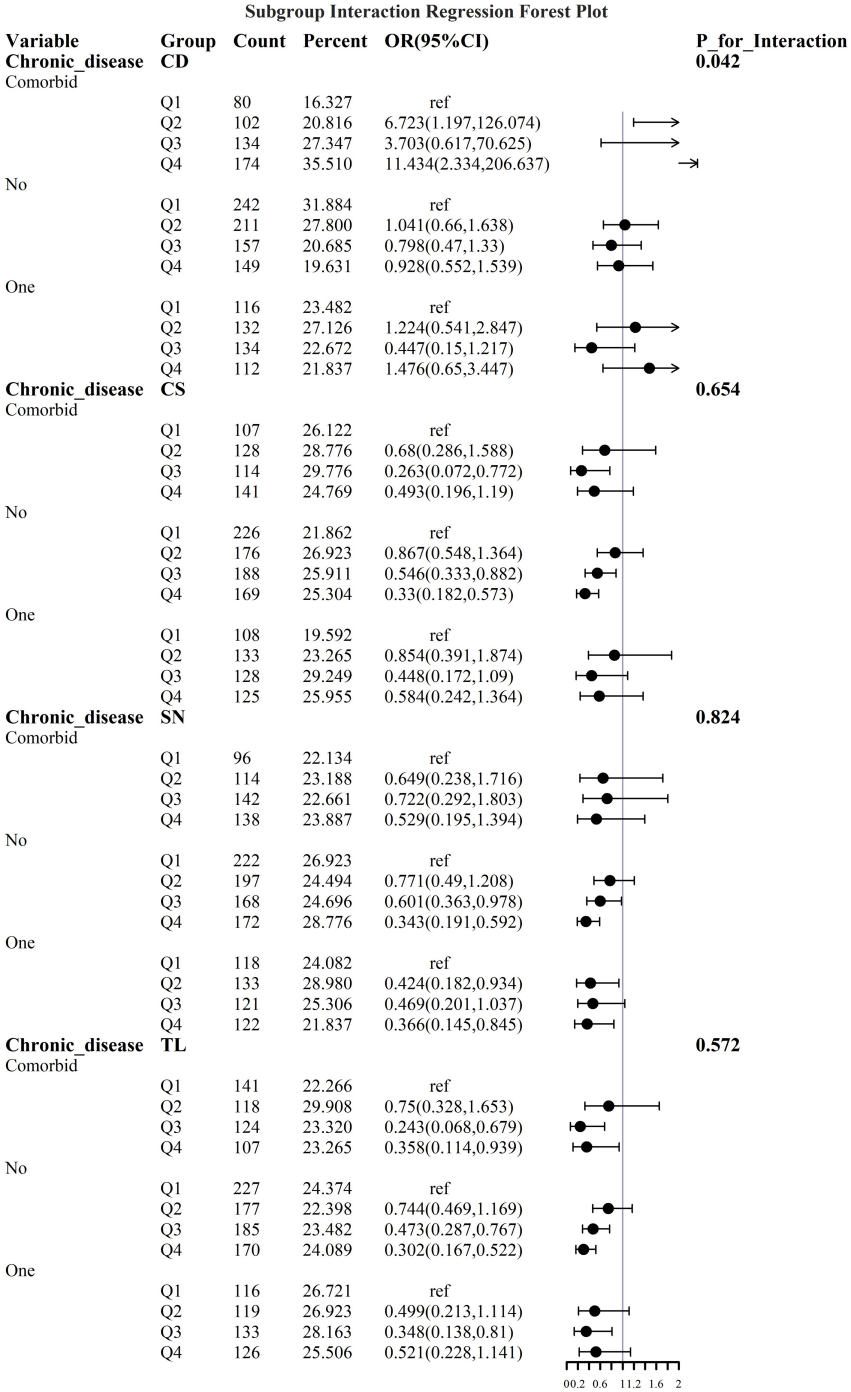
Logistic regression forest plots based on differences in the prevalence of chronic diseases.

To address the effects of confounding variables, we conducted interaction analyses for specific discussions. Starting with gender differences, Figure 10 illustrates a significant interaction between different gender groups regarding CS and SN, revealing that the association between CS and sarcopenia was significantly negative in females. The results presented in Figure 11 indicate that the interaction among different age groups was not significant for all variables (with *p*-values greater than 0.05 in all cases), suggesting that there is no meaningful difference in response or effect among various age groups concerning these variables. Regarding racial differences, we found that the association between heavy metal exposure and sarcopenia varied significantly across races (see Figure 12). Notably, non-Hispanic blacks exhibited a heightened response to heavy metal exposure, with the response at Q4 demonstrating a significant negative correlation with exposure levels of TL, CS, and SN. Figure 13 depicts a significant interaction between dietary factors (Hei) and CS levels, indicating a negative correlation. Furthermore, when analyzing chronic disease factors, we discovered that varying levels of chronic disease exposure significantly increased the risk of sarcopenia in populations with multiple comorbidities (see Figure 14). Reason: The revisions enhance clarity, improve vocabulary, and correct grammatical errors while maintaining the original meaning of the text.

## 4 Discussion

In this study, we utilized data from samples within the NHANES database spanning 2011 to 2018 to develop a ML model that integrated demographic characteristics (age, gender, ethnicity, and educational background), health factors (Le8 score and 14 chronic diseases), and components of heavy metal exposure to predict the risk of developing sarcopenia. Based on logistic regression analyses, we identified significant correlations between BA, CD, CS, PB, SN, TL, and sarcopenia. Among the six ML models evaluated, the LGBM model proved to be the most effective for predicting the risk of sarcopenia associated with heavy metal exposure. Through SHAP analysis with the LGBM model, we discovered that TL, SN, and CS contributed negatively to the risk prediction of sarcopenia, while CD contributed positively. Although the direct effects of heavy metal exposure on sarcopenia have not been clearly established in existing studies, it is important to consider the indirect pathways involved. The pathogenesis of sarcopenia also encompasses the effects of oxidative stress, inflammation, protein synthesis, and the nervous system. Heavy metal exposure has the potential to trigger these mechanisms, which are discussed in detail.

TL is the heavy metal predicted to have the greatest contribution to sarcopenia. Research indicates that TL does not directly cause significant pathological damage to skeletal muscle and bone (33). However, it can affect the nervous system, leading to sensory and motor alterations. Additionally, toxicity associated with TL has been linked to the production of reactive oxygen species and mitochondrial dysfunction. Secondly, there are effects on inflammation. A longitudinal experiment found that exposure to TL triggers the production of inflammation (34). In a study examining the impact of heavy metal exposure on sarcopenia, it was noted that inflammation induced by heavy metal exposure mediates sarcopenia (35). Thus, TL affects sarcopenia through complex mechanisms. In animal experiments, chronic ingestion of small amounts of TL has been found to yield results similar to those observed in humans who are chronically exposed to small amounts of TL, leading to damage and mitochondrial changes in the neuronal cells of the chondrogenic system (36). Exposure to indium tin oxide nanoparticles (ITO NPs) associated with systemic inflammation (SN) resulted in diffuse inflammatory infiltration of brain tissue, increased glial cell reactivity, abnormal neuronal lineage transformation, impaired neuronal migration, and neuronal apoptosis linked to oxidative stress (37). Low-dose exposure to heavy metals may enhance cellular autophagic activity by regulating the expression of autophagy-related genes (38). Heavy metal exposure leads to a reduction in mitochondrial membrane potential, subsequently disrupting normal mitochondrial function and inducing apoptosis. Additionally, it results in an increase in reactive oxygen species (ROS) within the mitochondria. These ROS not only compromise the structure and function of the mitochondria but also initiate intracellular oxidative stress, exacerbating cellular damage. Furthermore, mitochondrial dysfunction releases mitochondrial DNA (mtDNA), which activates intracellular inflammatory signaling pathways and promotes the production of inflammatory factors. Heavy metal exposure may also contribute to other chronic diseases that mediate sarcopenia. An empirical study has found a negative correlation between blood cadmium levels and lung function parameters (39). Chronic exposure to CD leads to renal tubular dysfunction. CD toxicity severely impacts cardiac health and induces significant biochemical and physiological changes. Furthermore, overexposure to CD is strongly associated with lung damage. All of these chronic conditions have been linked to sarcopenia in existing studies. This indicates a significant association between TL, SN, CS, CD, and sarcopenia, which can be utilized to predict the onset of sarcopenia. In the subgroup analyses, we found that the interaction between age and different heavy metal exposure environments yielded non-significant results. Cruz-Jentoft et al. noted that the magnitude of the effect of age on muscle mass (*β* = −0.4 to −0.6 SD/decade) was significantly greater than that of other risk factors, which may lead to a modification effect of environmental exposure that is difficult to detect (7). At the molecular level, mitochondrial damage in aged muscle has reached a plateau, and additional heavy metal stress may not produce distinguishable additive effects (40).

The le8 BMI also plays a significant role as a covariate in predicting the risk of sarcopenia. The le8 BMI is derived from the categorical coding of Body Mass Index (BMI). According to the established criteria, a score closer to the normal BMI standard indicates a higher score, while a lower le8 BMI score. It has demonstrated that low BMI is associated with a higher incidence of sarcopenia (41), and that as BMI decreases, the risk of sarcopenia increases (42). It has been found that the prediction of sarcopenia may be invalidated in overweight and obese populations due to the detection of individual-specific factors (43). Thus, leaner individuals have a greater risk of developing sarcopenia. The relationship between aging, one of the major factors in the development of sarcopenia, and sarcopenia is strong, but due to its confounding influence by multiple factors and the specificity of the human body, the effect of age, a covariate, on sarcopenia is more complex and needs to be discussed relying more on more dimensions. le8 pa and le8 smoke provide a negative contribution to the prediction of sarcopenia. Physical activity played a role in reducing the risk of sarcopenia (44), whereas there was an association between smoking and the onset of sarcopenia, with gradients presenting a 5% increase in sarcopenia and a 6% increase in severe sarcopenia for an additional 1 cigarette per day. However, this high level of association is broad but imprecise (45).

This paper has the following limitations: (i) data from the NHANES database from 2011–2018 are analyzed with a lag for such nationwide surveys, despite the older timeframe of radiation. The NHANES surveys, although they have been sampled to the greatest extent possible from the national level, the special geographic areas such as remote areas, minorities, and concentrations of people with specific occupational exposures are poorly represented, leaving out groups that may be at high risk of exposure. (ii) a cross-sectional study design was used, which made it impossible to capture the dynamic process of changes in heavy metal exposure levels and sarcopenia-related indicators over time. It only presents the association between the two at a static point in time, making it difficult to determine whether heavy metal exposure triggers sarcopenia or whether sarcopenic patients are more susceptible to heavy metal contamination due to changes in their physical functioning, making it impossible to conclusively determine a causal relationship. The urine exposure indicator reflects recent exposure rather than a long-term chronic body burden. There is an urgent need to conduct prospective cohort studies or introduce methods such as time series analysis to explore the causal chain. (iii) the LGBM models performed well on the NHANES data, and although some prediction accuracy was achieved after repeated debugging on the existing datasets, the generalization ability of these models needs to be further tested. In the future, more robust and explanatory composite models can be constructed by combining multiple machine learning algorithms with traditional statistical analyses, while newer and more diverse datasets are continuously introduced to improve the scientific validity and accuracy of the prediction of heavy metal exposure and sarcopenia risk in an all-round way. (iv) Although we adjusted for the primary lifestyle covariates, there may be measurement errors since these factors were primarily obtained through questionnaires. Additionally, some potentially relevant factors (e.g., sleep quality, micronutrient intake) were not measured, which may have resulted in residual confounding. This limitation could have impacted the accuracy of our estimated exposure-outcome associations. Future studies should consider utilizing more precise measurement tools (e.g., accelerometers, dietary records) and a more comprehensive collection of covariates to minimize such biases.

## 5 Conclusion

The best LGBM model was developed and selected using data from samples of individuals with sarcopenia in the NHANES database, and the model was interpreted using SHAP. Our analysis revealed that TL, SN, CS, and CD were associated with the risk of developing sarcopenia. Specifically, TL, SN, and CS were found to have a negative impact on risk prediction, while CD contributed positively to the risk assessment. Additionally, le8 BMI was identified as a significant covariate in this context. Furthermore, a strong correlation was observed between heavy metal exposure and the risk of sarcopenia, indicating that its inclusion in sarcopenia prediction models holds substantial practical value for identifying this condition. The ML model offers guidance for the development of sarcopenia prediction and presents new opportunities for identifying the risk of sarcopenia. Future studies should further investigate the relationship between other potential heavy metal exposures and sarcopenia, analyze their roles in muscle metabolism, neurotransmission, inflammation, and other pathophysiological processes, and elucidate the specific molecular mechanisms that contribute to the increased risk of sarcopenia.

## Data Availability

The original contributions presented in the study are included in the article/Supplementary material, further inquiries can be directed to the corresponding author.

## Footnotes

^1^ https://www.cdc.gov/nchs/nhanes/about/erb.html
