# Supplementary material for: Associations between exposure to heavy metal and sarcopenia prevalence: a cross-sectional study using NHANES data
Source: Front Public Health. 2025 Jul 4;13:1588041. doi: 10.3389/fpubh.2025.1588041 (PMC12272888; doi:10.3389/fpubh.2025.1588041)
Supplement: Supplementary file 3 [file Table_3.docx]

**Appendix**

Table S1: Abbreviations and full names used in articles

| Abbreviations | Full name |
| --- | --- |
| Le8 | Life’s essential 8 |
| RF | Random Forest |
| GBDT | Gradient Boosting Decision Tree |
| Logistic | Logistic Regression |
| LGBM | Light Gradient Boosting Machine |
| CatBoost | Categorical Boosting |
| AUC | Area Under the Receiver Operating Characteristic Curve |
| MCC | Matthews Correlation Coefficient |
| SHAP | SHapley Additive exPlanations |
| ML | Machine Learning |
| SMOTE | Synthetic Minority Over-sampling Technique |
| NHANES | National Health and Nutrition Examination Survey |

Table S2: Comparison table of variable names and descriptions

| Variable | Descriptions |
| --- | --- |
| Le8 BMI | Body Mass Index, BMI |
| Le8 pa | Physical Activity |
| Le8 hei | Healthy Eating |
| Le8 sleep | Machine Learning |
| Le8 smoke | Tobacco Exposure |
| Le8 bp | Blood Pressure |
| Le8 glucose | Blood Glucose |
| Le8 non hdl | Blood Lipids(Non-High-Density Lipoproteinemia) |
| BA | Barium |
| CD | Cadmium |
| CO | Cobalt |
| CS | Cesium |
| MN | Manganese |
| MO | Molybdenum |
| PB | Lead |
| SB | Antimony |
| SN | Tin |
| TL | Thallium |
| W | Tungsten |

Table S3: Lasso regression eigenvalue identification result.

| Coef Name | coeff_lamda |
| --- | --- |
| (Intercept) | -4.27579572136981 |
| sex | 0 |
| age | 0.00594744770664971 |
| race | 0.158683543958428 |
| edu | -0.0314732905810241 |
| BA | 0 |
| CD | 0.118757289388525 |
| CO | 0 |
| CS | -0.0308584071690545 |
| MN | 0 |
| MO | 0 |
| PB | 0 |
| SB | 0 |
| SN | -0.145351953180601 |
| TL | -0.266985409697884 |
| W | 0 |
| Chronic disease | -0.190587183653402 |
| le8 | 0 |
| le8 hei | 0 |
| le8 pa | -0.184890095948655 |
| le8 smoke | -0.0992392010975584 |
| le8 sleep | 0 |
| le8 BMI | 1.71976863155096 |
| le8 non hdl | 0.0949999244747929 |
| le8 glucose | 0 |
| le8 bp | 0 |

Table S4: Boruta Importance Scores and Boruta Decision.

| Variable Name | mean Imp | median Imp | min Imp | max Imp | norm Hits | decision |
| --- | --- | --- | --- | --- | --- | --- |
| le8 BMI | 34.2350479437786 | 34.5167297474301 | 28.8955105670878 | 38.9677538723908 | 1 | Confirmed |
| race | 10.4880726446511 | 10.4561783593636 | 7.21637065017387 | 14.6112465447215 | 1 | Confirmed |
| TL | 9.75970822637023 | 9.67387007049308 | 6.26339953874307 | 12.6114880246861 | 1 | Confirmed |
| CS | 8.79961507981367 | 8.8080531403632 | 5.0423242138542 | 11.0279505975764 | 1 | Confirmed |
| SN | 8.15518448196123 | 8.24591367923043 | 5.8024705673938 | 10.6712874718675 | 1 | Confirmed |
| CD | 7.79154487579312 | 7.72572518304792 | 5.19296296385318 | 11.3232943769062 | 1 | Confirmed |
| le8 | 6.50501043936372 | 6.46931599190904 | 3.53624596831994 | 8.3900642139408 | 1 | Confirmed |
| le8 pa | 6.49257072265532 | 6.46394883160845 | 4.41632991263352 | 9.84575724779551 | 1 | Confirmed |
| MO | 5.90851078854986 | 5.77067629559921 | 3.2194717921192 | 9.02867247348995 | 0.98989898989899 | Confirmed |
| age | 5.66075706008196 | 5.62257072968157 | 2.62968899602245 | 8.49894987813918 | 0.98989898989899 | Confirmed |
| le8 smoke | 5.44076522087091 | 5.50941673811506 | 2.6383697067872 | 7.96925290442518 | 0.98989898989899 | Confirmed |
| CO | 5.35591932319657 | 5.37479778110584 | 1.9700644064662 | 8.414418934077 | 0.96969696969697 | Confirmed |
| PB | 4.32592737300092 | 4.18208127937263 | 2.27512976269535 | 7.0523088467253 | 0.939393939393939 | Confirmed |
| le8 glucose | 4.03211479460293 | 3.99037511927845 | 1.30519444538488 | 7.14452666938793 | 0.898989898989899 | Confirmed |
| SB | 2.97164038533614 | 2.94669397886551 | -0.487600274809138 | 5.42198457501488 | 0.646464646464647 | Confirmed |

Table S5: Model Accuracy Statistics for Bootstrap Method Evaluation

| Model Name | Accuracy |
| --- | --- |
| LGBM_BR1TEST | 0.9334889148191365 |
| LGBM_BR2TEST | 0.9189031505250875 |
| LGBM_BR3TEST | 0.9107351225204201 |
| LGBM_BR4TEST | 0.9305717619603268 |
| LGBM_BR5TEST | 0.9311551925320887 |
| Mean | 0.9249708284714119 |
| Variance | 9.541166234823641e-05 |
| 95lower | 0.9054350497719859 |
| 95upper | 0.9445066071708379 |
